# Incidental discovery of circle contact lens by MRI: you can’t scan my poker face, circle contact lens as a potential MRI hazard

**DOI:** 10.1186/1471-2342-13-11

**Published:** 2013-03-25

**Authors:** Hiroyuki Tokue, Ayako Taketomi-Takahashi, Azusa Tokue, Yoshito Tsushima

**Affiliations:** 1 Department of Diagnostic and Interventional Radiology, Gunma University Hospital, 3-39-22 Showa-machi, Maebashi, Gunma 371-8511, Japan

**Keywords:** Circle contact lenses, Color contact lenses, Magnetic resonance imaging (MRI), Susceptibility artifact

## Abstract

**Background:**

Circle contact lenses, also known as color contact lenses and big eye contact lenses, are a type of cosmetic contact lens. It is not generally known that a circle contact lens usually contains iron oxide and other metals, which means their use during magnetic resonance imaging (MRI) is a potential hazard.

**Case presentation:**

We present a rare case of incidental discovery of circle contact lenses by MRI and MRI images of circle lenses in vitro.

**Conclusions:**

Circle contact lenses usually contain iron oxide, which is a known source of susceptibility artifact on MRI. Not only radiologists and radiographers but also referring physicians should be familiar with the imaging findings and potential risk of scanning circle contact lenses by MRI.

## Background

Circle contact lenses, also known as color contact lenses and big eye contact lenses, are a type of cosmetic contact lens. Circle contact lenses make one's eyes appear larger and come in a variety of colors and effects. They have received rather limited attention outside the Asian population until recently, but trends such as music videos of doll eyed pop stars have, reportedly, led to their increased use [1]. It is not generally known that a circle contact lens usually contains iron oxide and other metals [2], which means their use during MRI is a potential hazard.

We present a rare case of incidental discovery of circle contact lenses on MRI. Additionally, we recreated the image of circle contact lenses in vitro.

## Case presentation

**Figure 1:**
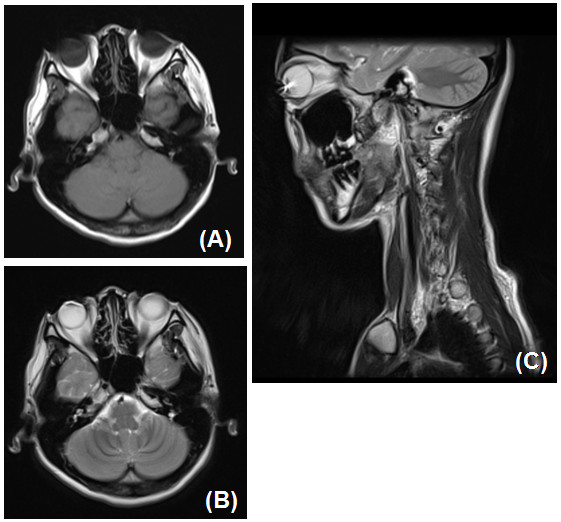
**A 28-year-old woman who wore circle contact lenses.** MRI showed a susceptibility artifacts in her eyes. **A**. T1-weighted fast spin-echo axial image: TR/TE, 545/15 (msec). **B**. T2-weighted fast spin-echo axial image: TR/TE, 4130/83 (msec). **C**. T2-weighted PROPELLER sagittal image: TR/TE, 3000/83 (msec). TR: repetition time, TE: echo time, PROPELLER: Periodically Rotated Overlapping Parallel Lines with Enhanced Reconstruction.

A 28 year-old woman who had a cervical desmoid underwent a follow-up MRI. MRI showed an artifact in the globes (Figure 1). She had experienced no discomfort or local heat or pain in her eyes during the examination. We asked her about her eyes, and it was proved that she wore circle contact lenses while being imaged in the 3.0 Tesla (3.0 T) MRI scanner (Magnetom Trio, Siemens Medical Systems, Erlangen, Germany). One year after this examination, she has not had any eye symptoms.

### In vitro study

We placed circle contact lenses [2-hydroxyethyl methacrylate (HEMA) with iron oxide] in a gelatin phantom and obtained T1 weighted images (WI), T2WIs and T2*WIs in our institution’s clinical 3.0 T MRI scanner. The diameter of the circle contact lenses was 14.2 mm, and the thickness was 0.08 mm. We measured a temperature change of circle contact lenses after MRI scan.

## Methods

### MR imaging protocol

T1-weighted fast spin-echo images were acquired using the following parameters: repetition time (TR), 600 msec; echo time (TE), 10 msec; flip angle, 90°. T2-weighted fast spin-echo images were acquired using the following parameters: TR, 4000 msec; echo TE, 80 msec; flip angle, 90°. T2*-weighted gradient-echo images were acquired using the following parameters: TR, 680 msec; TE, 20 msec; flip angle, 90°. For all sequences, matrix was 256 × 256; Field of view (FOV) 220 × 220 mm; number of excitations (NEX), one; section thickness, 3 mm.

## Results

**Figure 2:**
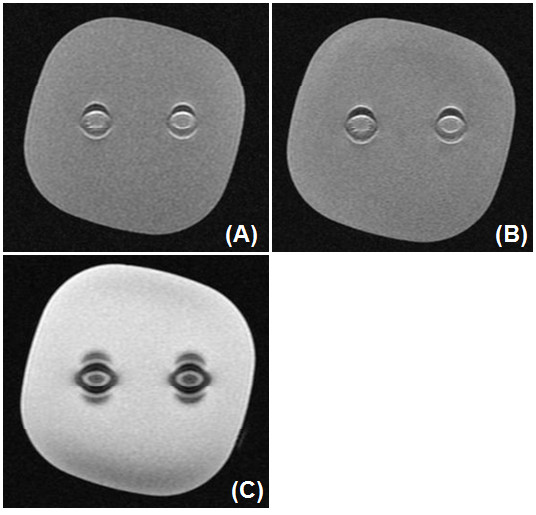
**MRI of gelatin phantom containing circle contact lenses showed susceptibility artifacts consistent with extra-wide outer rim of the lens. A**. T1-weighted fast spin-echo image: TR/TE, 600/10 (msec). **B**. T2-weighted fast spin-echo image: TR/TE, 4000/80 (msec). **C**. T2*-weighted gradient-echo image: TR/TE, 680/20 (msec).

MRI of the gelatin phantom containing circle contact lenses showed susceptibility artifact consistent with extra-wide outer rim of the lenses (Figure 2). The circle contact lenses was not deformed nor moved after the MRI scan. The total MRI scanning time was 8 minutes. After the MRI scan, the temperature of circle contact lenses rose 1.0 degree Celsius (°C).

## Discussion

Circle contact lenses have gained popularity in the younger population for their tendency to enlarge and draw attention to the iris. The diameter of regular contact lenses is similar to the diameter of the cosmetic circle contact lenses. The difference between the two types of lenses is that circle contact lenses are tinted not only in the area that covers the iris of the eye, but also has a rim slightly outside this area, usually in a darker color. The result is the appearance of a bigger, wider iris, which creates an illusion of large, doll-like eyes [1]. However, the extra-wide outer rim of the lens usually contains iron oxide, a known source of susceptibility artifact in MRI scans [3]. Indeed, the circle contact lenses showed susceptibility artifact consistent with extra-wide outer rim of the lenses in our in vitro case. However, the resolution of the clinical scanner used to image the circle contact lenses in vitro (0.86 mm in plane, 3 mm slice) was larger than the thickness of the circle contact lenses (0.08 mm), so that the images in Figure 2 might show the extent of the susceptibility artifacts rather than the circle contact lenses themselves.

There have been no reports in the English literature about the imaging findings of circle contact lenses in vitro and in vivo on MRI. There was only one Japanese report about the interactions of MR equipment with circle contact lenses [4]. They reported that a circle contact lens was not likely to be affected by the power of absorption by 3.0 T MRI. However, they did not verify the extent of temperature change during MRI scan.

Clinical MRI services should be aware of circle contact lenses as a source of decreased image quality as well as the theoretical risk of burns to the globe [5]. The cosmetic effects of circle contact lenses are much more subtle than the intended audience of computer graphics-enhanced music videos might be led to believe, and it is in fact sometimes difficult to tell they are being worn. Information on the content ratio of iron or other metals in circle lenses has not been made public. Several types of colored contact lenses are available and some of manufactures have revealed the contents, to some extent. Some colored contact lenses do not contain iron oxide. Currently, products of unknown origins may be also distributed. Removal of all contact lenses prior to an MRI examination should continue to be enforced, as it seems the only realistic way to prevent patients from being scanned while still wearing circle lenses.

## Conclusions

We presented a rare case of incidental discovery of circle contact lenses on MRI and the MRI imaging finding of circle lenses in a phantom. Not only physicians but also radiographers and referring physicians should be familiar with the imaging findings and potential risk of scanning circle contact lenses by MRI.

### Consent

Written informed consent was obtained from the patient for publication of this Case report and any accompanying images. A copy of the written consent is available for review by the Editor of this journal.

## Competing interests

The authors declare that they have no competing interests.

## Authors’ contributions

HT participated in the design of the study and carried out the clinical examination. All authors read and approved the final manuscript.

## Pre-publication history

The pre-publication history for this paper can be accessed here:

http://www.biomedcentral.com/1471-2342/13/11/prepub
